# Interaction of BMI and respiratory status in obstructive sleep apnea, a cross-sectional COPD study

**DOI:** 10.1038/s41533-023-00351-w

**Published:** 2023-08-15

**Authors:** Mizuha Haraguchi Hashiguchi, Shotaro Chubachi, Wakako Yamasawa, Kengo Otsuka, Naoko Harada, Naoki Miyao, Hidetoshi Nakamura, Koichiro Asano, Kazuhiro Yamaguchi, Koichi Fukunaga

**Affiliations:** 1 Internal Medicine, Keiyu Hospital, Yokohama-shi, Kanagawa Japan; 2 Internal Medicine, Nippon Koukan Hospital, Kawasaki-shi, Kanagawa Japan; 3 Division of Pulmonary Medicine, Department of Medicine, Keio University School of Medicine, Shinjyuku-ku, Tokyo Japan; 4 Department of Laboratory Medicine, Keio University School of Medicine, Shinjyuku-ku, Tokyo Japan; 5 Department of Respiratory Medicine, Saitama Medical University, Iruma-gun, Saitama Japan; 6 Division of Pulmonary Medicine, Department of Medicine, Tokai University School of Medicine, Sagamihara-shi, Kanagawa Japan; 7 Department of Pulmonary Medicine, Tokyo Medical University, Shinjuku-ku, Tokyo Japan

**Keywords:** Medical research, Epidemiology

## Abstract

This cross-sectional study of 136 patients with chronic obstructive pulmonary disease (COPD) investigated the mechanism underlying overlap syndrome, defined as coexisting COPD and obstructive sleep apnea (OSA). OSA was defined as a respiratory event index (REI) ≥ 5 events/h, determined using type-3 portable monitors. The mean REI was 12.8 events/h. Most participants (60.1%) had mild OSA (REI: 5–15 events/h). The REI was positively correlated with forced expiratory volume in one second (%FEV_1_) (*r* = 0.33, *p* < 0.001), body mass index (BMI) (*r* = 0.24, *p* = 0.005), and fat-free mass index (*r* = 0.31, *p* = 0.005), and negatively correlated with residual volume divided by total lung capacity (*r* = −0.27, *p* = 0.003). Receiver-operating characteristic curve analysis revealed an optimal BMI cutoff of 21.96 kg/m^2^ for predicting moderate/severe OSA. A BMI ≥ 21.96 kg/m^2^ was associated with OSA among participants with %FEV_1_ ≥ 50%, but not those with %FEV_1_ < 50%. This study revealed an interaction between airflow limitation and hyperinflation, nutritional status, and OSA.

## Introduction

The term “overlap syndrome” was originally introduced in 1985 to describe the association between two disorders, namely chronic obstructive pulmonary disease (COPD) and obstructive sleep apnea (OSA)^1^. Epidemiological studies have indicated a highly diverse prevalence of this syndrome, ranging from 1–3% in the general population to >60% in COPD cohorts^2^. To date, the clinical importance of overlap syndrome has increased due to its association with an increased risk of pulmonary hypertension, COPD exacerbation, and death^3^. Overlap syndrome is frequently undiagnosed^4^. To investigate the appropriate method of diagnostic evaluation and treatment of OSA, it is necessary to elucidate the associated risk factors and pathologies.

Recently, Zhu et al. reported that the apnea hypopnea index (AHI) was inversely correlated with airflow limitation^5^. Although the mechanisms of this phenomenon have not been clarified, COPD-elicited hyperinflation may improve apneas and hypopneas in overlap syndrome through the hyperinflation-elicited increase in stiffness at the pharyngeal site, which precludes the extrathoracic upper airway from collapsing^6^.

Moreover, cachexia is a major comorbidity in advanced COPD^7^, COPD-related hyperinflation and emphysema are associated with cachexia and muscle wasting^8^, and COPD patients with low muscle mass (as measured by the Fat Free Mass Index, FFMI) have a higher mortality^7^. Therefore, the task force for nutritional assessment recommended an evaluation of body composition for the clinical management of COPD. In contrast, obesity is the most common risk factor for obstructive sleep apnea (OSA), especially in Caucasians^9^. In consequence, the relationship between obesity and the severity of overlap syndrome remains controversial. The link between severity of COPD and sleep apnea, and the nutritional status of the patient is expected to be complex.

While polysomnography (PSG) is widely accepted as the gold standard for the diagnosis of OSA, the procedure is complicated, time-consuming, expensive, and not readily available in most hospitals. Recently, portable monitoring equipment comprising at least four channels (airflow, respiratory movements, oxyhemoglobin saturation, and heart rate) has been classified as a type-3 portable monitor and has been used as an alternative diagnostic test for OSA in high-risk patients with OSA, and more recently, their usefulness in COPD patients has been reported^10^ as well as their cost effectiveness^11^. Especially in the primary care setting, the usefulness of a type-3 portable monitor for diagnostic evaluation in outpatients is reported^12^.

Hence, we hypothesized that both physiological abnormalities of the lung and body composition might be associated with OSA, determined using portable sleep testing. The aims of the current study were to determine whether airflow limitation and/or hyperinflation and nutritional status, are associated with increased or decreased apnea and/or hypopnea formation using type-3 portable monitors.

## Methods

### Study population

This study was conducted at the Keio University and Nihon Kokan Hospital in Japan. The inclusion criteria for this cross-sectional study were: (1) age>40 years; (2) lifelong cigarette consumption≥10 pack-years; (3) COPD diagnosis; and (4) forced expiratory volume in one second/forced vital capacity ratio (FEV_1_/FVC) < 70%. The exclusion criteria were as follows: (1) severe systemic diseases, including malignancy in any organ, severe heart failure, or recent history of unstable myocardial infarction or stroke; (2) exacerbations or changes in medications in at least three months prior to study entry; and (3) prescription of long-term oxygen therapy. Sleep patterns and lung function were cross-sectionally examined in 136 patients with COPD. The research protocol was approved by the respective human ethics committees of the two institutions at Keio University School of Medicine (reference number: 2009–0008), and Nihon Kokan Hospital (reference number: 2015-2). All participants provided written informed consent.

### Sleep studies

The patients underwent unattended home monitoring for abnormal respiratory events during sleep with a type-3 portable device (Morpheus, Teijin Home Healthcare, Tokyo, Japan). The portable device recorded chest and abdominal respiratory movements, nasal air pressure, oxyhemoglobin saturation (SpO_2_), heart rate, and body position. The results were manually converted to scores by trained technicians based on the recommendations proposed by the American Academy of Sleep Medicine (AASM). Apnea was diagnosed based on the criteria that there was a drop in the respiratory signal by ≥90% of the pre-event baseline that lasted for ≥10 s. Hypopnea was measured based on the criteria that there was a drop in the respiratory signal by ≥30% but <90% of pre-event baseline in combination with ≥3% oxygen desaturation. Patients with a respiratory event index (REI, representing the sum of apnea and hypopnea events) above 5 events/h were defined as having overlap syndrome and compared with those without overlap syndrome (REI < 5 events/h).

### Lung function tests

All participants underwent spirometry and measurement of lung volume by helium dilution under stable conditions during baseline examination. Spirometric tests, including forced vital capacity (FVC), forced expiratory volume in one second (FEV_1_), forced expiratory flow-volume curve, and forced inspiratory flow-volume curve, were conducted using an electric spirometer (HI-801, CHEST MI, Tokyo, Japan). Residual volume (RV) and total lung capacity (TLC) were measured using the multi-breath helium dilution method. The value of diffusing capacity for carbon monoxide (CO) divided by the alveolar volume (DLCO/VA) was determined using the single breath method.

Maneuvers were performed according to the standardization of lung function testing recommended by the task force of the American Thoracic Society (ATS) and European Respiratory Society (ERS). Reference values for lung function parameters were calculated using equations reported by the Japanese Respiratory Society (JRS). The severity of airflow limitation was defined as grade 1 (FEV_1_ ≥ 80% of predicted value), grade 2 (FEV_1_ 50–80% of predicted value), grade3 (FEV_1_ 30–50% of predicted value) and grade 4 (FEV_1_ < 30% of predicted value) based on the 2017 Global Initiative for Chronic Obstructive Disease (GOLD) criteria.

To estimate extrathoracic upper airway collapsibility, the indicator of peak inspiratory flow rate (PIF) divided by peak expiratory flow rate (PEF) (PIF/PEF) was introduced^13,14^. The denominator and numerator on this indicator are in reverse order compared to the original FEF_50_/FIF_50_ indicator^15,16^ that has been used to evaluate extrathoracic airway obstruction in a functional sense. FIF_50_ corresponds to the forced inspiratory flow rate at 50% of inspiratory vital capacity (VC), and approximately corresponds to PIF in our equation. The usefulness of FEF_50_/FIF_50_ in subjects with no expiratory airflow limitation was extensively argued by many authors. Instead of FEF_50_, we adopted PEF in this study because FEF_50_ is crucially influenced by the intrathoracic peripheral airway obstruction, which is remarkably exaggerated in COPD. Hence, we considered that the PIF/PEF indicator might be better suited than FEF_50_/FIF_50_ and could act as a surrogate measure for extrathoracic upper airway collapsibility in lungs with COPD pathologies. A higher PIF/PEF implies a diminished collapsibility of the extrathoracic upper airway.

### Questionnaire and other measurements

At enrollment, full medical and smoking history, Epworth sleepiness scale (ESS), COPD assessment test (CAT), St. George’s Respiratory Questionnaire (SGRQ), and information on the current pharmacologic treatment and exacerbations were obtained.

Standing height and body weight were measured, and bioelectrical impedance analysis was performed using the BC309 (TANITA, Tokyo, Japan) to assess body composition, fat mass index (FMI), and fat-free mass index (FFMI). The study participants were divided into four groups to evaluate the mutual effects of airflow limitation and nutritional status modulation on apnea and hypopnea formation: Group 1 (BMI < 21.96 kg/m^2^ and %FEV_1_ < 50%), Group 2 (BMI ≥ 21.96 kg/m^2^ and %FEV_1_ < 50%), Group 3 (BMI < 21.96 kg/m^2^ and %FEV_1_ ≥ 50%), and Group 4 (BMI ≥ 21.96 kg/m^2^ and %FEV_1_ ≥ 50%). The cutoff value for BMI was calculated as the BMI that best predicts moderate/severe OSA.

Furthermore, based on the history of exacerbations and CAT scores, patients were divided into three groups of A, B, and E with GOLD classification 2023^17^: Group A is defined as a group with few subjective symptoms (CAT scores <10) and few exacerbations (one or fewer exacerbations not requiring hospitalization); Group B is a group with strong subjective symptoms (CAT score ≥10) but few exacerbations; and Group E is a group with many exacerbations (two or more exacerbations or at least one exacerbation requiring hospitalization) regardless of subjective symptoms.

### Statistical analysis

Data were compared among three or more groups using the Kruskal-Wallis and Steel-Dwass tests. The Jonckheere-Terpstra trend test was used to evaluate secular trends of the REI between the four groups stratified according to the Global Initiative Chronic Obstructive Lung Disease (GOLD) grade. Correlations between continuous variables were evaluated using the Pearson’s correlation coefficient. Receiver operating characteristic (ROC) curves were constructed to assess the areas under the curve (AUC). The optimal cutoff value was investigated by maximizing the Youden index. All calculations were performed using the IBM SPSS package (Version 22.0, IBM SPSS Inc., New York, NY, USA). The values are expressed as mean ± SD. Statistical significance was set at *P* < 0.05.

### Reporting summary

Further information on research design is available in the Nature Research Reporting Summary linked to this article.

## Results

### Prevalence of overlap syndrome

The patient characteristics are shown in Table 1. The mean age was 72.8 ± 7.8 years. At baseline, 28.7%, 43.4%, 25.0%, and 2.9% of the patients were diagnosed with GOLD grades 1, 2, 3, and 4, respectively. The mean REI was 12.8 events per hour and 22.8%, 47.1%, 22.8%, and 7.4% of the patients had a normal (REI < 5), mild (5 ≤ REI < 15), moderate(15 ≤ REI < 30), and severe (REI ≥ 30) OSA status, respectively. The mean ESS score was 4.2 ± 2.4, and only one patient had an ESS > 10 and was considered sleepy.

**Table 1:** Baseline clinical characteristics of study subjects.

	*N* = 136
Female (%)	12 (8.8)
Age (years)	72.8 ± 7.8
BMI (kg/m^2^)	22.8 ± 3.5
Cigarette consumption (pack-year)	57.3 ± 33.3
FEV_1_/FVC (%)	52.5 ± 12.3
FEV_1_ (ml)	1729 ± 655
FEV_1_ (% predicted)	65.8 ± 21.5
GOLD grades^a^ 1/2/3/4	39/59/34/4
DLCO/VA (% predicted)	67.3 ± 23.0
RV (ml)	1925 ± 587
RV (% predicted)	102.4 ± 31.5
RV/TLC	0.37 ± 0.08
PIF/PEF	0.60 ± 0.26
REI (events/hour)	12.8 ± 10.2
OSA severity^b^ Non/Mild/Moderate/Severe	31/64/31/10
ESS	4.2 ± 2.4
SGRQ total score	25.9 ± 17.6
CAT total score	11.8 ± 6.9

*Notes:* Data are shown as *n* (%), mean ± SD.

^a^Defined by the Global Initiative for Chronic Obstructive Lung Disease criteria: 1; FEV_1_ ≥ 80% of predicted value; 2, FEV_1_ 50–80% of predicted value; 3, FEV_1_ 30–50% of predicted value; 4, FEV_1_ < 30% of predicted value.

^b^Defined by AASM criteria: Non; REI < 5, Mild; 5 ≤ REI < 15, Moderate; 15 ≤ REI < 30, Severe; 30 ≤ REI

*BMI* body mass index, *GOLD* Global Initiative for Chronic Obstructive Lung Disease, *REI* respiratory event index, *OSA* obstructive sleep apnea syndrome, *ESS* Epworth Sleepiness Scale, *SGRQ* St. George’s Respiratory Questionnaire, *CAT* COPD assessment test, *AASM* American Academy of Sleep Medicine.

### Comparison of clinical characteristics of patients grouped according to the severity of sleep apnea

The characteristics of participants discriminated by REI values are shown in Table 2. Body mass index (BMI) was significantly different (*p* = 0.022) and FFMI was inclined to be different (*p* = 0.063) between the three groups, and both parameters increased in parallel with the severity of OSA (*p* = 0.007 and 0.014, respectively). There were no differences in age, smoking status, and ESS across the three groups.

**Table 2:** Comparison of baseline characteristics stratified by apnea and hypopnea severity.

	REI < 5	5 ≤ REI < 15	REI ≥ 15	
	*N* = 31	*N* = 64	*N* = 41	
Female (%)	6 (19.4)	4 (6.3)	2 (4.9)	*P* = 0.061
Age (year)	70.7 ± 8.5	73.0 ± 7.0	74.0 ± 8.3	*P* = 0.24
BMI (kg/m^2^)	21.8 ± 3.5	22.4 ± 3.5	24.0 ± 3.1	*P* = 0.022
FFMI (kg/m^2^)	17.2 ± 2.4	18.0 ± 1.6	18.7 ± 1.9	*P* = 0.063
Cigarette consumption (pack-year)	62.8 ± 28.5	59.2 ± 36.1	50.7 ± 33.3	*P* = 0.23
ESS	4.3 ± 2.4	4.2 ± 2.5	4.1 ± 2.5	*P* = 0.83
SGRQ total score	29.9 ± 15.9	23.8 ± 18.2	25.5 ± 18.2	*P* = 0.27
CAT score	13.1 ± 6.6	11.1 ± 6.9	11.5 ± 7.4	*P* = 0.45

*Notes:* Data are shown as n (%), mean ± SD.

*BMI* body mass index, *FFMI* fat free mass index, *ESS* Epworth Sleepiness Scale, *SGRQ* St. George’s Respiratory Questionnaire, *CAT* COPD assessment test.

### Relationships between airflow limitation, hyperinflation and apnea/hypopnea formation

**Fig. 1:**
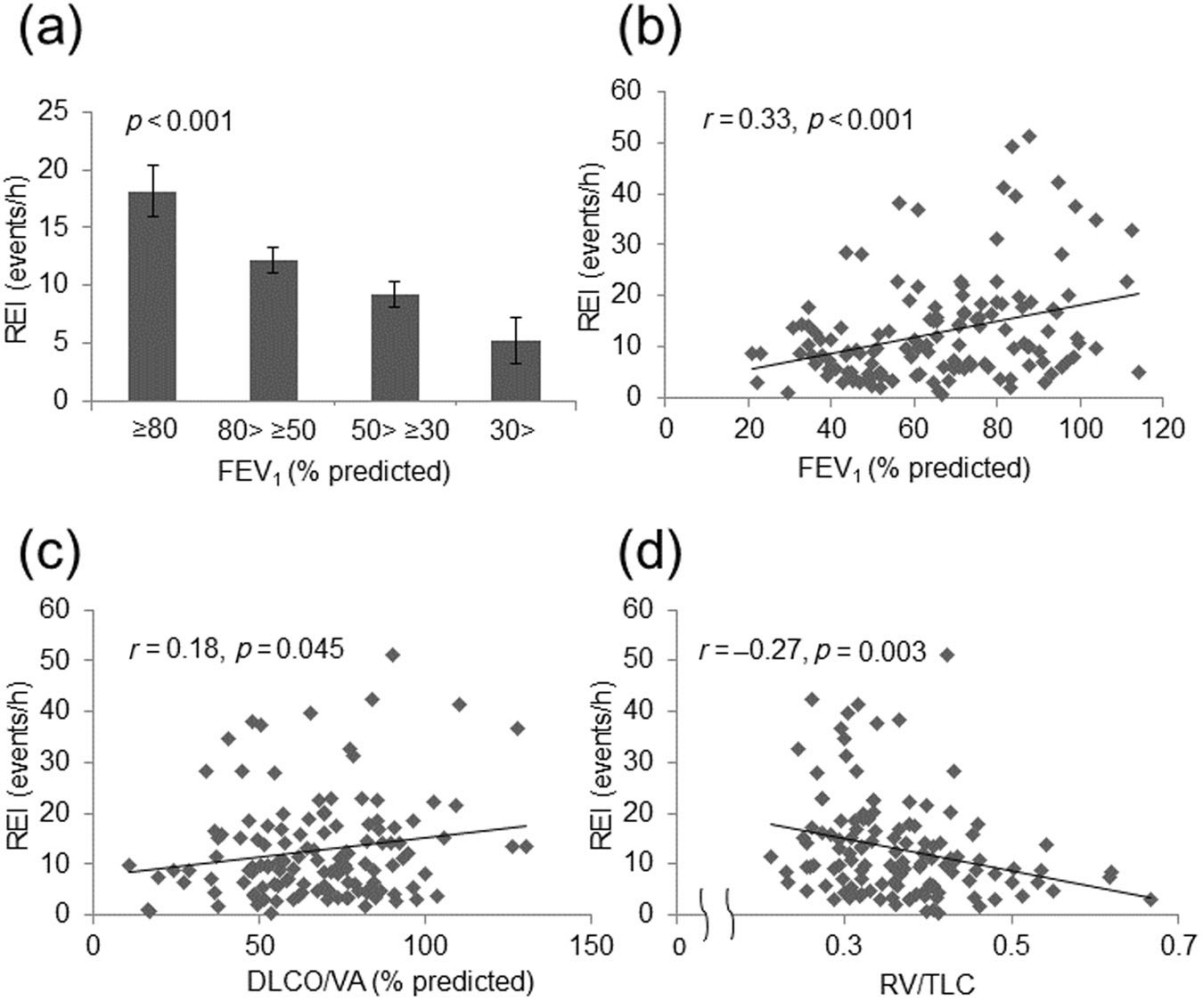
Relationships between apnea and hypopnea formation, and lung function. **a** Comparison of REI between the four groups stratified by the Global Initiative for Chronic Obstructive Lung Disease criteria: %FEV_1_ ≥ 80%; 80% > %FEV_1_ ≥ 50%; 50% > %FEV_1_ ≥ 30%; 30% > %FEV_1_. Correlation between REI and %FEV_1_ **b**, %DLCO/VA **c**, and RV/TLC **d**. REI respiratory event index, RV/TLC residual volume divided by total lung capacity, %DLco/VA percent predicted diffusing capacity for carbon monoxide divided by the alveolar volume, FEV_1_ forced expiratory volume in one second.

**Fig. 2:**
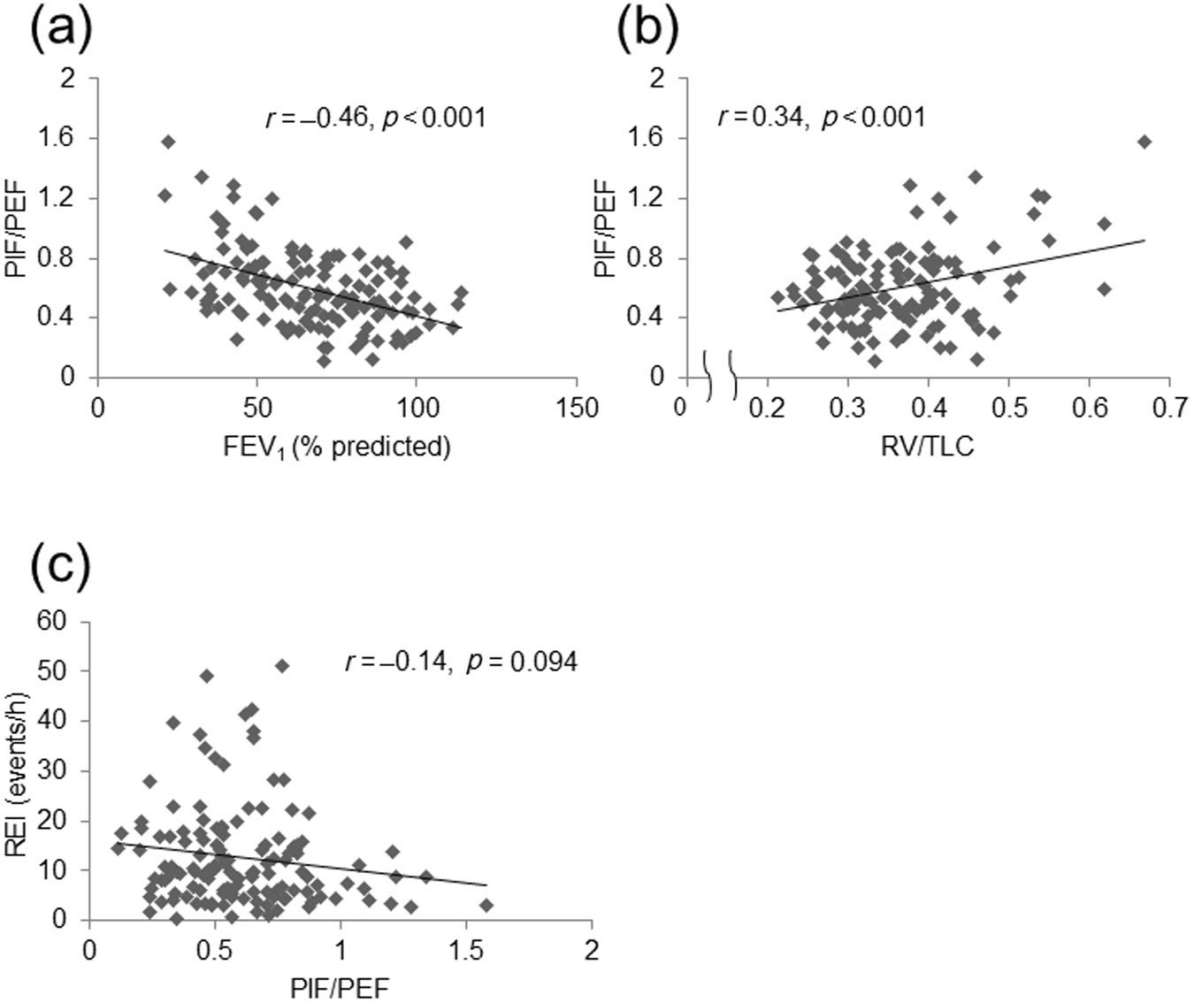
Correlation between PIF/PEF and lung function. Correlation between PIF/PEF and FEV_1_ **a**, RV/TLC **b**, and apnea and hypopnea formation **c**. REI respiratory event index, PIF/PEF peak inspiratory flow divided by peak expiratory flow, RV/TLC residual volume divided by total lung capacity, FEV_1_ forced expiratory volume in one second.

The relationships between the spirometric parameters and the REI are shown in Fig. 1. The comparison of REI stratified by FEV_1_ predicted value was shown in Fig. 1a. The REI decreased significantly with COPD severity. There was a significant correlation between FEV_1_ and the REI (*r* = 0.33, *p* < 0.001; Fig. 1b), and between DLco/VA and the REI (*r* = 0.18, *p* = 0.045; Fig. 1c). The hyperinflation-associated parameters, such as RV/TLC, were significantly correlated with the REI (*r* = -0.27, *p* = 0.003) (Fig. 1d). Evaluation of the relationships among airflow limitation, hyperinflation, and upper airway collapsibility was performed using the PIF/PEF ratio. PIF/PEF increased as airflow limitation (FEV_1_: *r* = -0.46, *p* < 0.001; Fig. 2a) and hyperinflation (RV/TLC: *r* = 0.34, *p* < 0.001; Fig. 2b) deteriorated. In addition, PIF/PEF tended to correlate with the REI (*r* = -0.14, *p* = 0.094) (Fig. 2c).

### Relationships between nutritional status and apnea/hypopnea formation

**Fig. 3:**
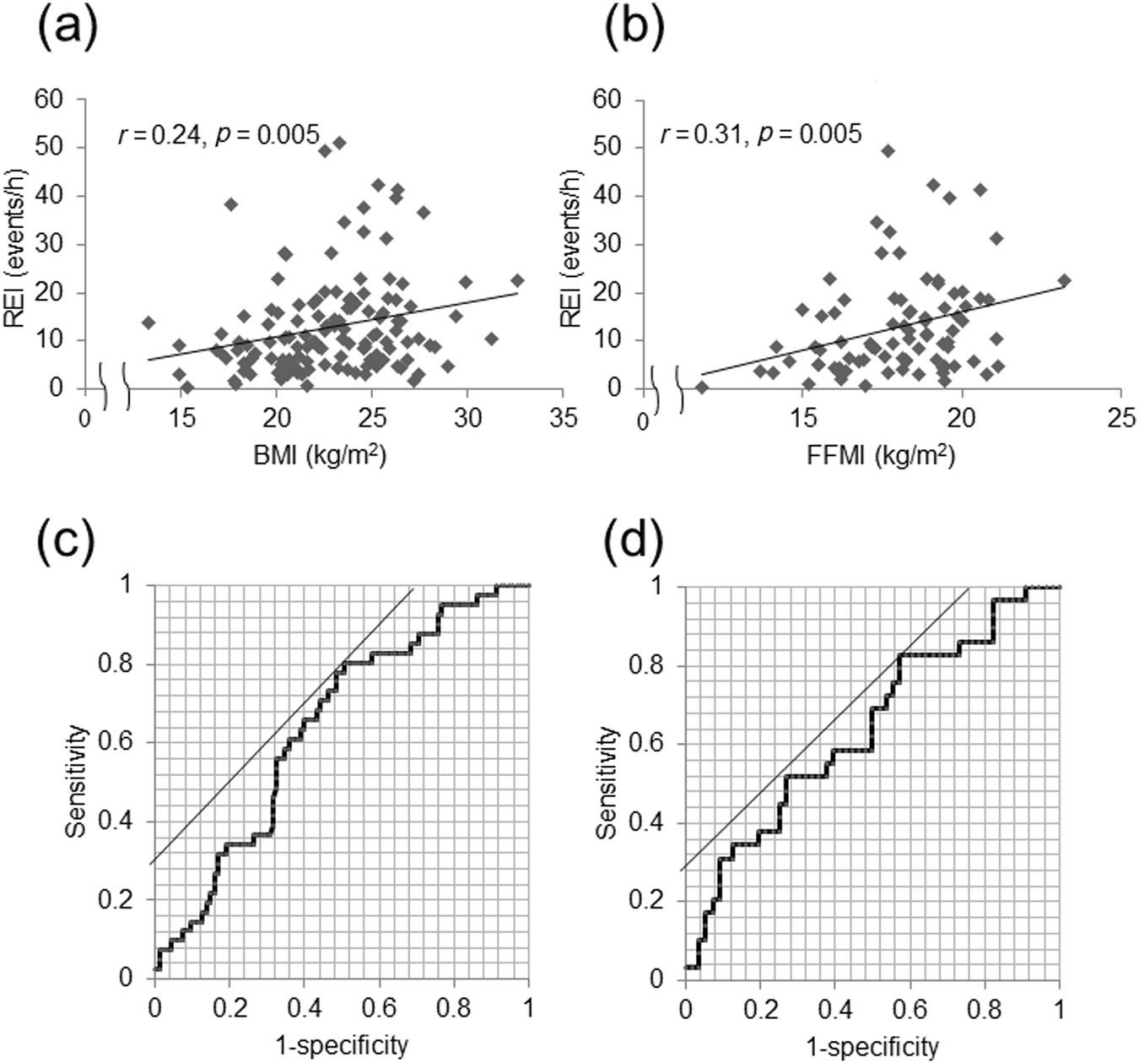
Relationships between hypopnea formation and nutritional status. Correlation between REI, and BMI **a** and FFMI **b**. Receiver operating characteristic curves of BMI **c** and FFMI **d** in predicting moderate/severe OSA. REI respiratory event index, BMI body mass index, FFMI fat-free mass index.

The BMI (*r* = 0.24, *p* = 0.005; Fig. 3a) and the FFMI (*r* = 0.31, *p* = 0.005; Fig. 3b) were significantly and positively correlated with the REI. ROC curves revealed that the optimal cutoffs for the BMI (AUC: 0.64, sensitivity: 80.5%, specificity: 49.5%; Fig. 3c) and the FFMI (AUC: 0.64, sensitivity: 82.8%, specificity: 42.9%; Fig. 3d) for predicting moderate/severe OSA were 21.96 and 17.30, respectively. In contrast with the association between FEV_1_ or RV/TLC and PIF/PEF, the BMI did not correlate with PIF/PEF (*r* = -0.08, *p* = 0.35).

### Comparison of apnea hypopnea index using classification of airflow limitation and BMI

**Fig. 4:**
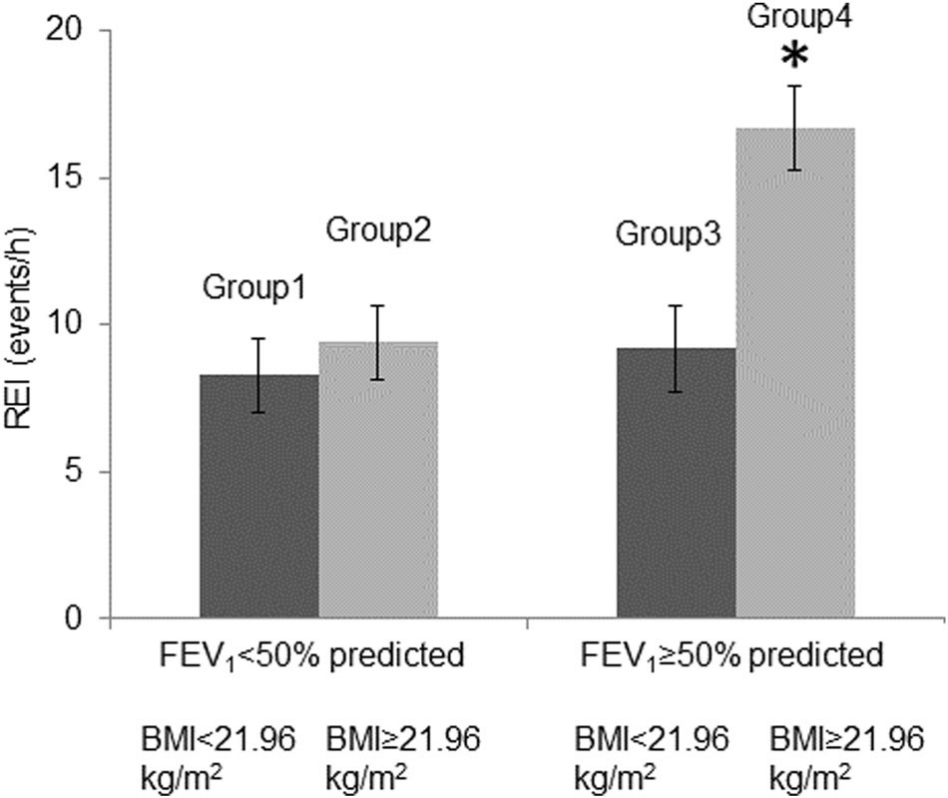
Comparison of apnea and hypopnea formation stratified by FEV_1_ and BMI. Group 1 (*n* = 24); BMI < 21.96 kg/m^2^ and FEV_1_ < 50 %predicted, Group 2 (*n* = 14); BMI ≥ 21.96 kg/m^2^ and FEV_1_ < 50% predicted, Group 3 (*n* = 31); BMI < 21.96 kg/m^2^ and FEV_1_ ≥ 50 % predicted, and Group 4 (*n* = 67); BMI ≥ 21.96 kg/m^2^ and FEV_1_ ≥ 50 % predicted. *Group 4 revealed a higher REI than groups 1, 2, and 3 (Group 4 vs. Group 1; *p* < 0.001, Group 4 vs. Group 2; *p* = 0.022, Group 4 vs. Group 3; *p* < 0.001). REI respiratory event index, BMI body mass index, FEV_1_ forced expiratory volume in one second.

The study participants were divided into four groups to evaluate the mutual effects of airflow limitation and BMI modulation on apnea and hypopnea formation: Group 1 (BMI < 21.96 kg/m^2^ and %FEV_1_ < 50%), Group 2 (BMI ≥ 21.96 kg/m^2^ and %FEV_1_ < 50%), Group 3 (BMI < 21.96 kg/m^2^ and %FEV_1_ ≥ 50%), and Group 4 (BMI ≥ 21.96 kg/m^2^ and %FEV_1_ ≥ 50%). Group 4 revealed a higher REI compared to Groups 1, 2, and 3 (Group1, 2, 3 vs. 4: *n* = 24, 14, 31, 67; REI = 8.3 ± 6.2, 9.4 ± 6.2, 9.2 ± 8.2 vs. 16.7 ± 11.4, *p* < 0.001, *p* = 0.022, *p* < 0.001 respectively; Fig. 4). On the other hand, there were no significant differences in REI between the three groups of A, B, and E based on the GOLD classification 2023 (Group A and B, vs. E: *n* = 32, 39, 8; REI = 13.0 ± 10.4, 12.7 ± 10.5, vs. 9.6 ± 8.8, *p* = 0.63; Supplemental Fig. 1).

## Discussion

This is a study to investigate the clinical characteristics of patients with COPD having OSA, determined using type-3 portable monitors. We believe that the results of the study are useful even in primary care settings, as PSG is often not available in primary care centers. In this study, type-3 portable monitors objectively revealed an OSA prevalence of 77.2% in Japanese patients with COPD. The observed prevalence is slightly higher than those reported previously^2^. The reason for this discrepancy is not clear, but it may reflect the different populations under investigation and the different methods or criteria for the diagnosis of overlap syndrome. As coexisting OSA worsens the prognosis of COPD patients^3^ and the survival of patients with overlap syndrome that is not treated with nocturnal positive airway pressure is significantly inferior to that of patients with overlap syndrome that is appropriately treated^3,18^, the diagnosis of OSA in COPD patients is important. However, overlap syndrome is frequently undiagnosed^4^. This study showed that OSA is a common comorbidity in Japanese patients with COPD. Two novel observations of potential relevance were made. First, the severity of airflow limitation, hyperinflation, and upper airway collapsibility were found to affect the REI in COPD patients through detailed spirometric assessment. Second the nutritional status, represented by BMI, is one of the most influential factors on the REI in COPD patients, even within the normal range.

Many factors related to coexisting COPD have been reported to modify the formation of apnea and hypopneas in overlap syndrome. Among them, decreased tethering of airways through the loss of lung recoil^19^, hyperinflation-augmented vagal-mediated reflex^20^, neural reflex-induced nasal obstruction^21^, and blunted responses of the respiratory center to various stimuli, particularly during REM sleep^22^, have been considered as supporting factors for apneas and hypopneas. On the other hand, hyperinflation-augmented stiffness of the upper airway walls^23^ is thought to be an important inhibitory factor against apnea and hypopnea formation. In fact, data from clinical trials revealed inverse relationships between AHI and FEV_1_ or radiological emphysema in patients with overlap syndrome^5,6^. The significant role of high lung volume in the inhibition of extrathoracic upper airway obstruction in subjects with no COPD was originally demonstrated by Spann and Hyatt in 1971^23^. Caudal traction through hyperinflation may unfold the airway or stiffen the airway walls to create a net ventral force that opens the extrathoracic upper airway, which leads to the inhibition of obstructive apnea and hypopnea formation (tracheal tug theory)^23–25^. Herein, we showed an important role of airflow limitation or TLC- and RV-related hyperinflation in restraining apnea and hypopnea formation. Our findings are consistent with the view that the hyperinflation-related inhibitory force against apnea and hypopnea formation is practically canceled out by the forces that promote apnea and hypopnea formation, including the decreased tethering of airways caused by the loss of elastic recoil in the destroyed lung parenchyma in patients with COPD.

The upper airway collapsibility provided by the PIF/PEF was associated with airflow limitation and hyperinflation, but the correlation between PIF/PEF and the REI was not significant (p = 0.09). Taken together, these findings suggest that although the PIF/PEF detects upper airway collapsibility under a specific condition, the PIF/PEF-represented upper airway opening force may be insufficient to alleviate the airflow limitation-related apnea and hypopnea formation, or the PIF/PEF does not reflect a dynamic change in upper airway collapsibility during sleep. No reports on the association between the PIF/PEF and other spirometric parameters in COPD patients are available. Therefore, to draw a definite conclusion regarding the effect of airflow limitation on upper airway collapsibility in association with apnea and hypopnea formation, it may be necessary to introduce a more precise method that allows the detection of dynamic changes in the upper airway cross-sectional area during sleep, such as CT^26^, MRI^27^, fluoroscopy^28^, or video-assisted nasoendoscopy^29^. However, these methods present difficulty in preserving natural sleep when applied to a sleeping subject.

The BMI and FFMI were significantly correlated with the REI. These results were consistent with those of a previous study, which showed that obesity is the most common risk factor for OSA in the general population^9^. Even if the BMI was within the normal range, a BMI > 21.96 kg/m^2^ was a predictor of moderate to severe OSA in our COPD population, especially in the preserved lung function groups (FEV_1_ ≥ 50% of predicted value). The relationship between obesity and the REI of overlap syndrome patients remains controversial^3^. While the reason for this discrepancy is not known, ethnic differences may be partly responsible. Asians including Japanese are generally less obese than Westerners. Thus, the threshold of BMI to predict the occurrence of OSA may differ among countries. Further, several studies, including our previous study, have demonstrated that the cachexia and emphysema phenotypes are more common in Japanese patients with COPD than in Western patients with COPD^30^. Therefore, whether this examination applies to Westerners should be determined henceforth. According to the results of this study, high BMI was associated with high AHI in patients with mild obstructive disorders, but not in those with severe obstructive disorders. Asians who are generally less obese than Westerners have been reported to be at a risk for OSA even in mild obesity^31^. It can be deduced that even a mild increase in BMI in this study was associated with high AHI in the mild COPD group. On the other hand, nutritional modulation has no effect on apnea and hypopnea development among patients with advanced COPD having an FEV_1_ below 50% of the predicted value. There are two possible reasons for the lack of such an association in severe COPD. One is that the effects of obstructive dysfunction and hyperinflation were more strongly associated with AHI than with high BMI. Therefore, the association with BMI may not have been apparent. The other reason is that few cases with a high BMI were included in the severe COPD group, making it difficult to appreciate the differences. In previous studies, severe COPD has been associated with a lower BMI and FFMI^32,33^. In fact, in our study, only 10.3% (*N* = 14/136) of patients with severe COPD had a higher BMI > 21.96 kg/m^2^. To examine the exact group differences, it may be necessary to study an even larger number of GOLD 1–4 patients.

In addition to airflow obstruction, we also investigated the impact of CAT score and history of exacerbations. There were no differences in REI between the three groups of A, B, and E based on the GOLD classification 2023. These results indicate that it may be appropriate to first focus on the degree of airflow obstruction and the risk of OSA complications rather than on subjective symptoms and exacerbations in primary care settings. In this study, there was only a small number of patients in Group E. This result was consistent with previous studies that reported that Japanese patients with COPD have a lower frequency of exacerbations^34,35^; however, more extensive studies are needed. There are several limitations to the present study. First, this is a cross-sectional study with only a single time point evaluation. As such, no conclusion can be drawn regarding the causal relationship between the abnormality of spirometric parameters or nutritional status and OSA severity. Second, the proportion of women was relatively small (8.8%), and the average age of the participants was higher than in the studies conducted in Western countries. Third, the study population included outpatients of a relatively large hospital. Therefore, caution should be exercised while applying the results of our study to patients visiting a clinic. Further, the number of cases is relatively small, and further investigations, such as a multicenter study in a clinic or a population-based study, are needed to determine whether our findings are applicable to the Japanese population as a whole as well as to the world general population. Fourth, the mechanism of upper airway obstruction has not yet been determined, as it has not been possible to directly observe the pharyngeal area in otolaryngology; some inferences about the mechanism have been made based on indirect data. Finally, lung function tests were not performed under the post-bronchodilator condition, and tests to exclude asthmatic elements, such as FeNO and peripheral blood eosinophils, were also not performed. Therefore, COPD may have been overdiagnosed, and cases with a combined asthmatic component were not completely removed. Many studies have shown that asthma has a negative impact on sleep apnea^19,36–38^, and it is possible that asthma complications have some impact on the pathogenesis of sleep apnea. The present study also did not measure eosinophils or FeNO, nor did it confirm the degree of reversibility, as mentioned above. Therefore, the effect of asthma complications on the results needs to be further investigated.

In conclusion, In Japanese COPD patients, airflow limitation and lung hyperinflation had a significant impact on the severity of OSA, with higher severity leading to lower REI. Further, BMI and FFMI were correlated with REI, especially in the preserved lung function groups. These findings may be useful in identifying high-risk groups for OSAS and the pathogenesis of overlap syndromes.

### Supplementary information

Reporting Summary

Supplementary Figure 1

## Data Availability

The data that support the findings of this study are available from the corresponding author, S.C.
