# Supplementary material for: Interaction of BMI and respiratory status in obstructive sleep apnea, a cross-sectional COPD study
Source: NPJ Prim Care Respir Med. 2023 Aug 15;33:30. doi: 10.1038/s41533-023-00351-w (PMC10427682; doi:10.1038/s41533-023-00351-w)

Supply Fig 1 Comparison of apnea and hypopnea formation between the three groups stratified by GOLD 2023.

Abbreviations: REI, respiratory event index.

Supplemental Figure 1

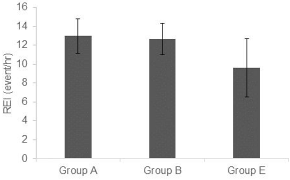

Supplement: Supplementary file 2 — Supplementary Figure 1 [file 41533_2023_351_MOESM2_ESM.pdf]
